# Temporal Clustering of Endometrial Cancer and Hyperplasia in HRT Users with Unscheduled Bleeding: A Retrospective NHS Cohort Study

**DOI:** 10.3390/curroncol33050284

**Published:** 2026-05-12

**Authors:** Mohamed Abdelwanis Mohamed Abdelaziz, Ahmed Mohamed, Ayodele Olaleye, Nesma Hesham, Nazifa Tasnim, Oluwafemi Ogundiran, Lorna Sandison, Hazem Sayed, Anita Juliana

**Affiliations:** 1 Department of Gynaecological Oncology, City Hospital, Nottingham University Hospitals NHS Trust, Nottingham NG5 1PB, UK; 2 Department of Gynaecology and Obstetrics, City Hospital, Nottingham University Hospitals NHS Trust, Nottingham NG5 1PB, UK; ahmed.mohamed28@nhs.net (A.M.); ayodele.olaleye@nhs.net (A.O.); nazifa.tasnim@nhs.net (N.T.); oluwafemi.ogundiran@nhs.net (O.O.); lorna.sandison@nhs.net (L.S.); hazem.sayed1@nhs.net (H.S.); 3 Department of Gynaecology and Obstetrics, Queen’s Medical Centre, Nottingham University Hospitals NHS Trust, Nottingham NG7 2UH, UK; nesma.hesham@nhs.net (N.H.); anita.juliana1@nhs.net (A.J.)

**Keywords:** endometrial cancer, hormone replacement therapy, unscheduled bleeding, postmenopausal bleeding, retrospective cohort, early detection, temporal clustering, diagnostic yield

## Abstract

**Background:** Unscheduled bleeding in postmenopausal women on hormone replacement therapy (HRT) represents a common but poorly characterised clinical challenge. Whilst endometrial cancer affects approximately 9% of women with unexplained postmenopausal bleeding, existing evidence in HRT users is largely restricted to women under 60 years and short-duration regimens, leaving a critical evidence gap in contemporary all-age clinical practice. Whether the same investigative urgency is warranted for HRT users experiencing unscheduled bleeding as for women with unexplained postmenopausal haemorrhage remains unresolved. **Objectives:** To determine the diagnostic yield of endometrial cancer and hyperplasia amongst postmenopausal women presenting with unscheduled bleeding whilst on HRT, and to explore potential associations with HRT regimens and clinical risk factors. **Methods:** This retrospective observational study analysed 343 postmenopausal women presenting with unscheduled bleeding whilst on HRT at a single tertiary centre between September 2023 and February 2024. All patients underwent transvaginal ultrasound and endometrial sampling according to institutional protocol. Descriptive statistics were used to characterise outcomes, with exploratory analyses of potential risk factors. Given the symptomatic and selected nature of this cohort, all proportions represent the diagnostic yield within an investigated population rather than population-level incidence estimates. **Results:** Amongst 343 women (mean age 56.2 ± 7.4 years), nine cases (2.6%) of endometrial abnormalities were identified: four malignancies (1.2%), four endometrial hyperplasia without atypia (1.2%), and one complex atypical hyperplasia (0.3%). Only five cases (1.5%) required surgical intervention. All four endometrial cancers were Stage IA (FIGO 2009; three Grade 1, one Grade 2; no LVSI), corresponding to Stage IA2mNSMP under FIGO 2023. None required adjuvant therapy. Strikingly, 88.9% of all abnormal cases occurred within two years of HRT initiation, with no endometrial pathology identified amongst the 45 women using systemic HRT for more than five years—a temporal pattern not previously reported. **Conclusions:** In this retrospective all-age NHS cohort, the diagnostic yield of endometrial cancer was 1.2% in HRT users with unscheduled bleeding, with only 1.5% requiring surgical intervention. All cancers were early-stage (FIGO 2009 Stage IA; FIGO 2023 Stage IA2mNSMP) and required no adjuvant therapy. A previously unreported temporal clustering of pathology within the first two years of HRT initiation generates a hypothesis that early unscheduled bleeding may unmask pre-existing rather than HRT-induced endometrial abnormalities. These observations are hypothesis-generating and should not be interpreted as evidence of endometrial safety. These findings apply specifically to symptomatic women presenting for investigation and cannot be generalised to asymptomatic HRT users. Prospective validation in larger cohorts with baseline endometrial assessment is required before any clinical conclusions can be drawn. **What This Study Adds:** (1) A real-world cancer detection proportion of 1.2% in an all-age contemporary NHS cohort. (2) A previously undescribed temporal pattern with pathology clustering within two years of HRT initiation and no pathology in long-term users (*n* = 45), generating a testable hypothesis about pre-existing versus HRT-induced disease. (3) Dual FIGO 2009/2023 staging demonstrating that molecular classification added no treatment-discriminatory value in this early-detection context.

## Simple Summary

Unscheduled bleeding in postmenopausal women using hormone replacement therapy (HRT) is a common clinical presentation. This study examined 343 women investigated for unscheduled bleeding during HRT use at a UK NHS centre. Endometrial cancer was detected in 1.2% and significant endometrial pathology in 2.6% of cases. All detected cancers were at the earliest possible stage and required surgery alone with no further treatment. A previously unreported temporal pattern was observed: the vast majority of abnormal findings occurred within the first two years of HRT use, with no pathology detected in women using HRT for more than five years (*n* = 45). These findings are hypothesis-generating and suggest that early unscheduled bleeding may unmask pre-existing endometrial abnormalities rather than represent de novo HRT-related disease. These data may inform patient counselling regarding the probability and characteristics of pathology detected. Prospective studies with pre-HRT baseline endometrial assessment are required to test this hypothesis.

## 1. Introduction

Postmenopausal bleeding (PMB) represents a significant clinical concern requiring prompt evaluation to exclude endometrial malignancy. Pooled evidence from over 40,000 patients indicates that endometrial cancer is identified in approximately 9% of women presenting with unexplained postmenopausal bleeding, underpinning current guideline recommendations for urgent investigation [1,2]. However, this widely cited estimate derives from heterogeneous populations and conflates women with truly unexplained bleeding with a clinically distinct subgroup: postmenopausal women using hormone replacement therapy (HRT) who develop unscheduled bleeding [3]. Whether these two groups carry equivalent endometrial cancer risk, and therefore whether they warrant identical investigative urgency, remains an unanswered clinical question with direct implications for service design and patient counselling.

The relationship between HRT and endometrial safety has evolved significantly with our understanding of hormone therapy regimens. Contemporary HRT protocols, particularly continuous combined preparations, were developed specifically to minimise endometrial stimulation and reduce the risk of endometrial hyperplasia and cancer [4,5]. The clinical relevance of this question has grown substantially: HRT prescriptions in England approximately doubled between 2018 and 2023 following updated NICE guidance and increased public awareness of menopausal health, meaning that unscheduled bleeding in HRT users is an increasingly common presentation in rapid-access gynaecology services [6].

The occurrence of endometrial pathology amongst HRT users presenting with unscheduled bleeding remains inadequately characterised. A prior UK single-centre study reported a cancer detection proportion of only 0.47% in HRT users with postmenopausal bleeding, but was restricted to women under 60 years of age and did not examine temporal relationships between HRT initiation and pathology detection, leaving older HRT users unaddressed [7]. Multiple factors potentially influence endometrial cancer occurrence in this population, including HRT regimen, duration of use, and patient characteristics such as BMI [5,8]. The Million Women Study demonstrated that continuous combined regimens carry endometrial-protective effects whilst unopposed oestrogen increases risk [9], yet how these regimen differences manifest in the symptomatic bleeding population has not been comprehensively examined.

Current clinical practice guidelines recommend investigation of all postmenopausal bleeding episodes, including those occurring during HRT use [10,11]. Currently, NICE NG12 classifies all postmenopausal bleeding as a two-week-wait referral without distinguishing HRT users from non-users [11]. If malignancy occurrence is substantially lower in the HRT-user subgroup, a more nuanced risk-stratified approach may be worthy of future evaluation without compromising cancer detection [12].

This exploratory observational cohort study aimed to describe and characterise the diagnostic yield of endometrial cancer and hyperplasia in a contemporary all-age NHS cohort of postmenopausal women presenting with unscheduled bleeding whilst on HRT, and to examine the temporal relationship between HRT initiation and pathology detection—an association not previously reported. Secondary objectives included characterising potential associations between endometrial pathology and HRT regimen type, endometrial thickness, and clinical risk factors, and applying dual FIGO 2009/2023 staging to all detected cancers. These analyses are hypothesis-generating in nature only.

## 2. Aim of Work

### 2.1. Primary Objective

To determine the diagnostic yield of endometrial cancer and hyperplasia amongst postmenopausal women presenting with unscheduled bleeding whilst on hormone replacement therapy.

### 2.2. Secondary Objectives

To explore the association between HRT regimen types (continuous combined vs. sequential) and endometrial pathology.

To evaluate the relationship between endometrial thickness on ultrasound and histopathological findings.

To examine the temporal relationship between HRT initiation and development of endometrial pathology.

### 2.3. Exploratory Analyses

Assessment of clinical risk factors (BMI, diabetes, family history) and their potential association with endometrial pathology.

Evaluation of diagnostic yield from different investigative approaches.

Examination of the temporal relationship between HRT initiation and timing of endometrial pathology detection.

## 3. Methods

### 3.1. Study Design and Setting

This retrospective observational study was conducted at Queen’s Medical Centre, Nottingham University Hospitals (NUH) NHS Trust, examining records of postmenopausal women who presented with unscheduled bleeding whilst on HRT between 1 September 2023 and 29 February 2024.

### 3.2. Study Population

All postmenopausal women (defined as absence of menstruation for ≥12 months) presenting with unscheduled bleeding whilst on any form of HRT during the study period were eligible. Exclusions: incomplete clinical or histopathological data, previous history of endometrial cancer, or use of tamoxifen or other selective oestrogen receptor modulators. Based on institutional historical data, 340–360 eligible cases were anticipated over the six-month period.

### 3.3. Data Collection

Data were extracted from electronic medical records using a standardised form. Parameters recorded: patient demographics (age, BMI, parity); HRT information (type and duration); clinical risk factors (family history of gynaecological cancers, diabetes mellitus); clinical investigations (endometrial thickness by transvaginal ultrasound, ultrasound setting, type of endometrial sampling, hysteroscopy findings); and pathological outcomes (histological diagnosis, grade of hyperplasia, endometrial cancer with FIGO staging). Missing data: BMI 11.9%, parity 13.2%, HRT duration 10.2%; analyses performed on available data without imputation.

### 3.4. Study Limitations Regarding Baseline Assessment

Baseline endometrial assessment prior to HRT initiation was not routinely performed in this cohort, consistent with current guideline-based clinical practice in the United Kingdom (NICE NG23 [6]), which recommends investigation only in symptomatic women. Routine pre-HRT transvaginal ultrasound or endometrial sampling is not recommended for asymptomatic women commencing HRT in UK practice. Where selective pre-treatment investigations were performed in women with pre-existing bleeding symptoms or high-risk features, these were not consistently documented in the retrospective records and therefore could not be incorporated into the analysis. This is an important limitation: without pre-HRT baseline data, it is not possible to determine definitively whether the abnormalities detected after HRT initiation were newly developed or pre-existing. The temporal findings must therefore be interpreted as hypothesis-generating only.

### 3.5. Clinical Pathways and Diagnostic Procedures

All patients were evaluated according to the institutional protocol aligned with RCOG guidelines [10]. Initial assessment included transvaginal ultrasound scanning (community or hospital), followed by endometrial sampling. Hysteroscopy was performed based on clinical indications, endometrial thickness measurements, and/or inconclusive sampling results.

### 3.6. Statistical Analysis

This study was descriptive and hypothesis-generating in design; not powered for definitive risk estimation, multivariable modelling, or causal inference. Continuous data presented as mean ± SD or median with IQR; categorical variables as frequencies and percentages. For exploratory analyses, Student’s *t*-test or Mann–Whitney U test were used for continuous variables and Chi-square or Fisher’s exact test for categorical variables. Multivariable regression analyses were not performed given limited events. Statistical analyses were performed using SPSS version 28.0 (IBM Corp., Armonk, NY, USA). *p*-values are presented descriptively and should not be interpreted inferentially.

## 4. Results

### 4.1. Study Population and Clinical Characteristics

During the six-month study period, 1399 postmenopausal women presented with postmenopausal bleeding at our centre. Of these, 343 women (24.5%) met inclusion criteria. The mean age was 56.2 ± 7.4 years; 28.6% were aged 60 years or older. Mean BMI was 29.2 ± 6.1 kg/m^2^ (range 17.5–53.0); 28.6% were obese (BMI ≥ 30). The majority (77.8%) were parous. Diabetes mellitus was present in 5.0%; family history of gynaecological cancers in 12.8% (Table 1).

### 4.2. HRT Usage Patterns

Continuous combined HRT was the most commonly used regimen (72.6%), followed by local vaginal oestrogen (9.9%) and sequential combined HRT (8.2%). Most patients had used HRT for less than 5 years (76.7%); 13.1% had used HRT for more than 5 years (Table 2). Specific HRT formulations and dosages were not consistently documented in the retrospective records; regimen categories as presented represent the level of granularity available for analysis.

### 4.3. Investigation Pathways

All patients underwent transvaginal ultrasound as the initial investigation (84.3% community settings). Endometrial sampling performed in all patients, predominantly via Pipelle biopsy (86.9%). Hysteroscopy performed in 145 patients (42.3%) (Table 3).

### 4.4. Primary Outcome: Endometrial Pathology

Histopathological analysis revealed nine cases (2.6%) with significant endometrial abnormalities: four cases of endometrial cancer (1.2%), four cases of endometrial hyperplasia without atypia (1.2%), and one case of complex atypical hyperplasia (0.3%). The remaining 334 cases (97.4%) showed normal or benign findings. These findings characterise the real-world diagnostic yield of current investigation pathways in this population.

Only five cases (1.5%) required surgical intervention (four endometrial cancers and one complex atypical hyperplasia), whilst the four cases of hyperplasia without atypia were successfully managed with medical therapy and HRT modification.

### 4.5. Characteristics of Patients with Abnormal Findings

The clinical characteristics of the nine patients with abnormal endometrial findings are detailed in Table 4 and Table 5. The mean age of patients with abnormal findings was 58.1 ± 10.5 years (range: 49–84 years). The mean BMI was higher in patients with abnormal findings compared to those with normal findings (33.1 ± 7.7 vs. 29.2 ± 6.1 kg/m^2^; interpreted cautiously given the small number of events).

### 4.6. FIGO Staging and Treatment Outcomes

All four patients with endometrial cancer underwent total hysterectomy with bilateral salpingo-oophorectomy.

### 4.7. Under FIGO 2009 Classification

Three cases: Stage IA Grade 1 endometrioid adenocarcinoma, no LVSI. One case: Stage IA Grade 2 endometrioid adenocarcinoma, no LVSI. All cases classified as LOW RISK according to ESGO/ESTRO/ESP guidelines. Treatment: surgery only, no adjuvant therapy required.

### 4.8. Under FIGO 2023 Classification

All 4 cases: Stage IA2mNSMP (low-grade endometrioid adenocarcinoma with <50% myometrial invasion, no LVSI, NSMP molecular profile). Treatment: surgery only, no adjuvant therapy required.

### 4.9. Temporal Relationship Between HRT Initiation and Pathology

A previously undescribed temporal pattern was observed: eight of nine cases (88.9%) with abnormal findings occurred in women who had been using systemic HRT for ≤2 years. The only exception was a patient using local vaginal oestrogen for 25 years. Amongst the 45 women who had used HRT for >5 years, no endometrial pathology was identified, though this subgroup is too small to exclude pathology reliably. This observed pattern is hypothesis-generating and may suggest that endometrial abnormalities in early HRT users represent pre-existing rather than HRT-induced pathology, though the absence of baseline endometrial assessment data prevents any definitive interpretation.

### 4.10. Exploratory Analysis of Potential Risk Factors

Given that only nine abnormal cases and four cancers were identified, all subgroup analyses are strictly hypothesis-generating; no causal inference is possible. The mean endometrial thickness was higher in patients with abnormal findings compared to those with normal findings (10.6 ± 2.9 mm vs. 7.9 ± 4.3 mm, *p* = 0.02). Sequential combined HRT users showed a higher proportion of abnormal findings compared to continuous combined HRT users (10.7% vs. 2.0%; unadjusted odds estimate 5.35, 95% CI: 1.35–21.20); this numerical trend should be regarded as hypothesis-generating only. Obesity (BMI ≥ 30) showed a higher proportion of abnormal findings (6.1% vs. 1.5%; unadjusted estimate 4.12, 95% CI: 1.05–16.13). Endometrial thickness > 10 mm showed a higher proportion (7.2% vs. 1.2%; unadjusted estimate 6.22, 95% CI: 1.59–24.32). All three estimates are statistically unstable due to small event numbers (Table 6).

### 4.11. Diagnostic Performance of Ultrasound Thresholds

Given the importance of endometrial thickness in risk stratification, we examined the diagnostic performance of different ultrasound thresholds. Using the traditional 5 mm cut-off would have identified all abnormal cases (100% sensitivity) but with low specificity (23.4%), resulting in many unnecessary invasive procedures. The 10 mm threshold showed better balance with 66.7% sensitivity and 77.2% specificity. However, three cases of pathology had endometrial thickness < 10 mm, including two malignant cases (5.9 mm and 9.2 mm), highlighting important limitations of any single threshold approach in HRT users. Clinical judgement incorporating multiple risk factors remains essential. 

## 5. Discussion

This study examined endometrial cancer diagnostic yield, disease characteristics, and temporal risk patterns in a contemporary all-age NHS cohort of postmenopausal women with HRT-related unscheduled bleeding. Two key observations merit further investigation: all four endometrial cancers were detected at early stage (FIGO Stage IA, NSMP molecular profile) requiring surgery alone with no adjuvant therapy; and a temporal clustering of pathology within the first two years of HRT initiation was observed. Both findings should be interpreted in the context of a small number of events and neither supports causal inference without prospective validation.

Our analysis identified nine cases (2.6%) with significant endometrial abnormalities, including four cases of endometrial cancer (1.2%). Only five cases (1.5%) required surgical intervention. This diagnostic yield is lower than the frequently cited 5–10% cancer proportion in women with unexplained postmenopausal bleeding [13,14], though this study is not sufficient to establish endometrial safety.

To our knowledge, this is among the first contemporary all-age NHS cohort studies to characterise endometrial cancer diagnostic yield specifically in HRT users with unscheduled bleeding, and the first to examine the temporal relationship between HRT initiation and pathology detection. Our 1.2% cancer detection proportion is higher than the 0.47% reported by Buchanan et al. in a UK rapid-access gynaecology clinic restricted to women under 60 years [7], a difference likely explained by our inclusion of all age groups: 28.6% of our cohort were aged 60 years or older. The low surgical intervention rate (1.5%) and conservative management of nearly half the abnormal cases (4/9) with progestogen therapy is relevant to counselling women considering HRT [15,16].

All four endometrial cancers received identical treatment recommendations under both FIGO 2009 and FIGO 2023 classifications. Whilst Ferrari et al. reported that FIGO 2023 re-staging reduced adjuvant treatment needs by 16% in broader early-stage cohorts [17], the additional discriminatory value of molecular classification appears limited in this specific context where all detected cancers were early-stage.

The predominant use of continuous combined HRT (72.6%) reflects current clinical practice. Our exploratory analysis suggested a potential association between sequential HRT use and endometrial pathology, consistent with larger studies showing better endometrial protection with continuous combined preparations [18,19]. This numerical trend is consistent with established evidence from the WHI and Cochrane reviews showing enhanced endometrial safety with continuous versus sequential preparations [5,20].

Notably, obesity was present in the majority of women with significant pathology (6/9, 66.7%), consistent with the established mechanistic link between adiposity and endometrial cancer risk through aromatase-mediated peripheral oestrogen production [9,21,22]. This numerical observation—though based on small absolute numbers and without multivariable adjustment—raises the hypothesis that endogenous oestrogen excess driven by obesity may be more clinically important than HRT regimen in some patients, and supports the argument that pre-HRT endometrial risk stratification in obese women may deserve specific consideration in future prospective work.

### 5.1. Temporal Relationship and Pre-Existing Pathology Hypothesis

To our knowledge, this is the first study to describe a temporal clustering of endometrial pathology in relation to HRT initiation in an all-age cohort of women with unscheduled bleeding. The observation that 88.9% of abnormal cases occurred within two years of starting systemic HRT, with no cases observed amongst the 45 long-term users (>5 years), raises the possibility that early unscheduled bleeding may serve as a symptomatic alert that unmasks pre-existing rather than HRT-induced endometrial disease. However, this subgroup is underpowered to exclude pathology.

We acknowledge that baseline endometrial assessment data prior to HRT initiation were not available in our cohort, consistent with standard UK guideline-based practice (NICE NG23 [6]), which recommends investigation only in symptomatic women. This prevents definitive confirmation of whether detected pathologies were pre-existing or HRT-induced. Several alternative explanations must be explicitly considered: (1) survivorship bias—women with pre-existing endometrial abnormalities may present with early bleeding and discontinue HRT; (2) detection bias—women presenting with early unscheduled bleeding may be more closely investigated; (3) reverse causation—subclinical endometrial pathology present before HRT initiation may itself contribute to early bleeding. These alternative explanations cannot be distinguished without prospective data including pre-HRT baseline endometrial assessment.

This finding contrasts with the Million Women Study, which suggested increasing endometrial cancer risk with longer duration of oestrogen-only HRT use [9]. However, our cohort predominantly used combined HRT regimens (80.8%), which may partly explain this difference.

### 5.2. Ultrasound Threshold Performance and Limitations

Importantly, in symptomatic women presenting with postmenopausal bleeding—including those using HRT—current clinical guidelines (RCOG Green-top Guideline No. 67 [10]; NICE NG12 [11]) recommend histological evaluation irrespective of endometrial thickness. The threshold analysis presented here is therefore descriptive and exploratory, contextualising current investigation practice within this cohort, and should not be interpreted as support for a threshold-based approach to deferring investigation in symptomatic women.

Our data suggest that traditional ultrasound thresholds for postmenopausal bleeding may not be directly applicable to HRT users [23,24]. The mean endometrial thickness in abnormal cases (10.6 mm) was higher than in normal cases (7.9 mm). Using the standard 5 mm threshold would have captured all abnormal cases (100% sensitivity) but with only 23.4% specificity. The 10 mm threshold showed better balance (66.7% sensitivity, 77.2% specificity) but would have missed three abnormal cases, including two malignancies (5.9 mm and 9.2 mm). A comprehensive approach incorporating HRT type, duration, and BMI alongside endometrial thickness is preferable to reliance on any single threshold.

### 5.3. Hysteroscopy Rate and Diagnostic Yield

The 42.3% hysteroscopy rate reflects indication-based selection in line with RCOG guidance [10]. The finding that 28.6% of our cohort were aged ≥60 years reflects contemporary individualised HRT prescribing guided by NICE and IMS frameworks, which no longer impose strict age limits [6]. The relatively small proportion of long-term users (13.1%) likely reflects the lasting prescribing caution following the WHI study [25].

### 5.4. Clinical Implications

The substantially lower cancer diagnostic yield (1.2%) compared to general postmenopausal bleeding populations (5–10%) raises the question of whether these two groups differ meaningfully in risk, though this requires prospective validation. These data have value in patient counselling: women who develop unscheduled bleeding whilst on HRT can be informed that malignancy was identified in only 1.2% of cases in this cohort, that 98.5% avoided surgical intervention, and that when cancer was detected it presented at its most treatable stage.

The intended clinical contributions of this study are three-fold. (1) Counselling utility: real-world diagnostic yield data applicable to patient counselling during shared decision-making about HRT. (2) Hypothesis generation: the temporal pattern generates a testable hypothesis about pre-existing versus HRT-induced pathology. (3) Future research design: candidate risk variables—sequential HRT use, obesity, and endometrial thickness > 10 mm—provide a basis for prospective study design. Asymptomatic HRT users are not routinely investigated in UK standard practice (NICE NG23 [6]); this study characterises the diagnostic yield in the clinically relevant symptomatic subgroup and should not be interpreted as providing evidence regarding endometrial safety in asymptomatic users.

## 6. Strengths and Limitations

This study is drawn from a consecutive series of 1399 postmenopausal bleeding presentations at a tertiary NHS centre, of whom 343 (24.5%) were HRT users, providing a real-world prevalence figure directly applicable to NHS rapid-access gynaecology services. Inclusion of all age groups enhances generalisability.

However, important limitations must be acknowledged.

(1) Absence of baseline endometrial assessment (primary limitation): Baseline endometrial assessment prior to HRT initiation was not routinely performed, consistent with NICE NG23-based UK practice. This is the most significant interpretive limitation. Without pre-HRT data, it is impossible to determine whether detected pathology was pre-existing or HRT-induced. Selective pre-treatment investigations, where performed, were not consistently documented.

(2) Single-centre design: Findings may not generalise to populations with different demographics or clinical protocols.

(3) Limited statistical power: With only four cancer cases and nine abnormal findings, the study was not powered for risk factor analysis. Confidence intervals are wide and no causal inference is possible.

(4) Retrospective design: We lack baseline endometrial assessment before HRT initiation, preventing definitive conclusions about whether pathology was pre-existing or HRT-related.

(5) Short follow-up: Median follow-up of 6 months limits ability to detect delayed diagnoses or recurrences.

(6) Missing data: 10–13% for key variables.

(7) Absence of comparator group: The absence of a comparator group of non-HRT users with postmenopausal bleeding prevents any inference regarding relative cancer risk attributable to HRT use. These findings apply specifically to symptomatic women presenting for evaluation and should not be extrapolated to the broader HRT-using population.

(8) HRT exposure detail: Detailed information regarding specific HRT formulations, dosages, and adherence was not consistently available, precluding formulation-level risk analysis.

(9) Selection bias: Women presenting with bleeding may differ from all HRT users.

(10) Ultrasound threshold limitations: The occurrence of malignancy below proposed thresholds highlights the limitations of any single diagnostic cut-off.

### Future Directions

A multicentre prospective cohort study incorporating mandatory pre-HRT baseline endometrial assessment (transvaginal ultrasound ± biopsy in selected cases) would constitute the most direct test of the temporal hypothesis generated by this dataset. Secondary objectives should include validation of the candidate risk variables—sequential HRT use, obesity (BMI ≥ 30), and endometrial thickness > 10 mm—in a cohort powered for multivariable logistic regression. A cost-effectiveness analysis of modified investigation pathways stratified by duration of HRT use is also warranted. Longer-term follow-up studies are required to ensure that conservatively managed hyperplasia without atypia does not progress.

## 7. Conclusions

In this exploratory observational cohort of 343 postmenopausal women with unscheduled bleeding on HRT, endometrial cancer was identified in 1.2% and significant endometrial pathology overall in 2.6% of cases—a diagnostic yield that appeared lower than rates reported for unexplained postmenopausal bleeding, though this comparison is indirect. Only 1.5% required surgical intervention, and all four cancers were early-stage (FIGO 2009 Stage IA; FIGO 2023 Stage IA2mNSMP), classified identically under both systems and requiring no adjuvant therapy. Unscheduled bleeding continues to warrant investigation in all cases.

The temporal pattern of pathology detection—with most abnormal cases occurring within two years of HRT initiation and none observed amongst long-term users (*n* = 45)—is a previously unreported observation that generates a testable hypothesis: early unscheduled bleeding may represent the unmasking of pre-existing endometrial abnormalities rather than de novo HRT-induced disease. This interpretation is one of several plausible explanations, including survivorship bias and selection bias, and cannot be confirmed without prospective data including pre-HRT baseline endometrial assessment.

Exploratory analyses identified numerical signals for sequential HRT use (unadjusted estimate 5.35), obesity (unadjusted estimate 4.12), and endometrial thickness > 10 mm (unadjusted estimate 6.22) as candidate variables for future risk stratification.

These findings are not intended to alter current investigation pathways. They may, however, provide useful data to inform patient counselling regarding the probability and characteristics of pathology detected, and generate hypotheses for future prospective research. Unscheduled bleeding continues to warrant investigation in all cases.

Prospective cohort studies incorporating pre-HRT baseline endometrial assessment are needed to test the temporal hypothesis, and multicentre studies are required to validate candidate risk variables before any modified investigation pathway could be considered.

## Figures and Tables

**Table 1:** Demographic and clinical characteristics of study population (*N* = 343).

Characteristic	Value
Age (years)	
Mean ± SD	56.2 ± 7.4
<50 years	47 (13.7%)
50–59 years	198 (57.7%)
60–69 years	76 (22.2%)
≥70 years	22 (6.4%)
BMI (kg/m^2^)	
Mean ± SD	29.2 ± 6.1
Range	17.5–53.0
Underweight (<18.5)	3 (0.9%)
Normal (18.5–24.9)	89 (25.9%)
Overweight (25–29.9)	112 (32.7%)
Obese (≥30)	98 (28.6%)
Not documented	41 (11.9%)
Parity	
Nulliparous	31 (9.0%)
Parous	267 (77.8%)
Not documented	45 (13.2%)
Risk Factors	
No risk factors	258 (75.2%)
Family history	44 (12.8%)
Diabetes mellitus	17 (5.0%)
Other risks	24 (7.0%)

**Table 2:** HRT types and usage patterns.

HRT Characteristic	*n* (%)
Continuous combined	249 (72.6%)
Sequential combined	28 (8.2%)
Local vaginal oestrogen	34 (9.9%)
Oestrogen-only	12 (3.5%)
Other (e.g., tibolone/combined types)	20 (5.8%)
Duration <1 year	89 (25.9%)
Duration 1–2 years	98 (28.6%)
Duration 2–5 years	76 (22.2%)
Duration >5 years	45 (13.1%)
Not documented	35 (10.2%)

Note: Specific HRT formulations and dosages were not consistently documented; regimen categories represent the level of granularity available for analysis.

**Table 3:** Investigation Settings and Procedures.

Investigation Characteristic	*n* (%)
Community ultrasound	289 (84.3%)
Hospital ultrasound	54 (15.7%)
Pipelle biopsy	298 (86.9%)
Other endometrial biopsy	45 (13.1%)
Hysteroscopy not performed	198 (57.7%)
Outpatient hysteroscopy	112 (32.7%)
Under general anaesthesia (GA)	33 (9.6%)

**Table 4:** Summary characteristics of patients with abnormal findings.

Characteristic	Cancer (*n* = 4)	Hyperplasia (*n* = 5)	*p*-Value *
Age (years)			
Mean ± SD	61.8 ± 15.6	55.2 ± 6.8	0.38
Range	49–84	49–65	
BMI (kg/m^2^)			
Mean ± SD	32.3 ± 13.1	33.8 ± 4.2	0.81
Range	20.3–46.0	28.0–40.0	
ET (mm)			
Mean ± SD	10.6 ± 4.6	10.7 ± 1.7	0.96
Range	5.9–15.5	8.2–12.0	

* *p*-value for comparison between cancer and hyperplasia groups.

**Table 5:** Detailed characteristics of cases with endometrial abnormalities.

Case	Age	BMI	Diagnosis	HRT Type	HRT Duration (Years)	ET (mm)	Other Risk Factors	Treatment	FIGO 2009	FIGO 2023
1	49	20.3	EC (G1)	Sequential	2	5.9	None	TH + BSO	IA	IA2mNSMP
2	57	46.0	EC (G2)	Continuous	2	9.2	Diabetes, Family Hx	TH + BSO + SLN Biopsy	IA	IA2mNSMP
3	84	28.8	EC (G1)	Local vaginal	25	15.5	None	TH + BSO	IA	IA2mNSMP
4	57	34.1	EC (G1)	Continuous	2	11.8	Obesity	TH + BSO	IA	IA2mNSMP
5	57	40.0	CAH	Sequential	1	8.2	Obesity	TH + BSO	N/A	N/A
6	49	28.0	EH w/o atypia	Continuous	2	10.3	None	Progestogen + FU	N/A	N/A
7	65	34.0	EH w/o atypia	Continuous	2	12.0	Obesity, Diabetes	Change to different continuous combined HRT+ FU	N/A	N/A
8	54	31.5	EH w/o atypia	Sequential	2	10.8	Obesity	Change from sequential to continuous combined HRT+ FU	N/A	N/A
9	51	33.3	EH w/o atypia	Continuous	1	11.5	Obesity	Change to different continuous combined HRT+ FU	N/A	N/A

EC: Endometrial Cancer. CAH: Complex Atypical Hyperplasia. EH: Endometrial Hyperplasia. TH: Total Hysterectomy. BSO: Bilateral Salpingo-Oophorectomy. SLN: Sentinel Lymph Node. FU: Follow-up. NSMP: Non-Specific Molecular Profile. G: Grade. ET: Endometrial Thickness. Hx: History. w/o: without.

**Table 6:** Exploratory analysis of potential risk factors for abnormal endometrial findings.

Risk Factor	Normal Findings	Abnormal Findings	Unadjusted Estimate (95% CI)
HRT Type			
Continuous combined (*n* = 249)	244 (98.0%)	5 (2.0%)	Reference
Sequential combined (*n* = 28)	25 (89.3%)	3 (10.7%)	5.35 (1.35–21.20)
Other (*n* = 66)	65 (98.5%)	1 (1.5%)	0.75 (0.09–6.30)
BMI Category			
Non-obese (*n* = 204)	201 (98.5%)	3 (1.5%)	Reference
Obese (BMI ≥ 30) (*n* = 98)	92 (93.9%)	6 (6.1%)	4.12 (1.05–16.13)
Endometrial Thickness			
≤10 mm (*n* = 260)	257 (98.8%)	3 (1.2%)	Reference
>10 mm (*n* = 83)	77 (92.8%)	6 (7.2%)	6.22 (1.59–24.32)

Note: Due to small numbers of events, these exploratory analyses should be interpreted with caution and considered hypothesis-generating rather than confirmatory. Important Note: All risk factor analyses were exploratory and hypothesis-generating. No adjustments were made for multiple comparisons.

## Data Availability

The datasets used and analysed during the current study are available from the corresponding author on reasonable request, subject to approval from the Nottingham University Hospitals NHS Trust Clinical Audit Department and in accordance with relevant data protection regulations. The data are not publicly available due to privacy and ethical restrictions.

## Abbreviations

BMI: Body Mass Index. HRT: Hormone Replacement Therapy. PMB: Postmenopausal Bleeding. NUH: Nottingham University Hospitals. NHS: National Health Service. RCOG: Royal College of Obstetricians and Gynaecologists. ET: Endometrial Thickness. EC: Endometrial Cancer. EH: Endometrial Hyperplasia. CAH: Complex Atypical Hyperplasia. TH: Total Hysterectomy. BSO: Bilateral Salpingo-Oophorectomy. GA: General Anaesthesia. FIGO: International Federation of Gynecology and Obstetrics. RR: Risk Ratio. CI: Confidence Interval. LVSI: Lymphovascular Space Invasion. NSMP: Non-Specific Molecular Profile. IMS: International Menopause Society. NICE: National Institute for Health and Care Excellence.
